# Significant Missed Polyps in the UK Bowel Cancer Screening Programme (BCSP): A Retrospective Analysis of Prevalence and Contributing Factors

**DOI:** 10.7759/cureus.72360

**Published:** 2024-10-25

**Authors:** Anita Golash, Kevin Yoong, Ramasamy Saravanan

**Affiliations:** 1 Gastroenterology, Macclesfield District General Hospital, Macclesfield, GBR; 2 Gastroenterology, Leighton Hospital, Crewe, GBR

**Keywords:** artificial intelligence, bowel cancer prevention, bowel preparation, colonoscopy, colorectal cancer screening, flat polyps, missed polyps, premalignant polyps

## Abstract

Background

Colorectal cancer (CRC) remains a significant public health challenge. Patients having abnormal faecal immunochemical test (FIT) results are offered a colonoscopy. The effectiveness of colonoscopies can, however, often be challenged by the occurrence of missed polyps. This study aims to assess the rate of significant missed polyps in the Bowel Cancer Screening Programme (BCSP) in the UK.

Methods

A retrospective analysis of BSCP screening data in the Cheshire region in the UK from 2020 to March 2023 was conducted. A significant polyp was defined as a polyp ≥ 10mm. The inclusion criteria included patients (age range: 54-74 years) who had had an index colonoscopy followed by site checks, repeats, or planned polypectomies.

Results

Out of 2,759 index colonoscopies, 261 (9.5%) met our criteria, and 23 (8.8%) of these had significant polyps. Of the 261, the missed polyp rate was 30% (453/1531 polyps). The overall significant missed polyp rate was 1.6% (24/1531). Of the missed polyps, 5% (24/453) were significant polyps. The majority (71%) of the significant polyps were found on the left of the colon. Men had a higher missed polyp rate (22%) compared to women (7%) (relative risk (RR) = 2.56, 95% CI: 2.1-3.13, p<0.0001). They also had a higher significant missed polyp rate (1.1%) compared to women (0.4%) (RR = 2.41, 95% CI: 1-5.8, p<0.05). A total of 50% of the bowel prep at index colonoscopy was rated as ‘adequate/fair’ and 79% of the bowel prep at the discovery of the significant polyp was rated as either ‘excellent’ or ‘good' (odds ratio (OR) = 3.8, 95% CI: 1.07-13.5, p<0.05); 92% (22/24) of the significant polyps found were either tubular adenoma (TA) low-grade dysplasia (LGD) or tubular villous adenoma (TVA) LGD, and none were found to be cancerous.

Conclusions

Almost a third of all polyps detected were missed, and one in 20 of these were significant polyps, putting these patients in the high-risk group for CRC. Improving bowel preparation and monitoring patients with multiple polyps could reduce the rate of missed polyps.

## Introduction

Colorectal cancer (CRC) remains a significant public health concern. It is the third most common cancer diagnosis and the second leading cause of cancer-related deaths. In 2020, approximately 9.4% of all cancer deaths were linked to CRC [1]. Another concerning aspect is that the prevalence of CRC is estimated to double by 2035, particularly in developing nations [2]. In the United Kingdom (UK), approximately 44,000 cases of CRC are diagnosed each year [3]. In a significant number of cases, CRC develops from premalignant polyps [4]. Although not all polyps turn into cancer, polyps greater than or equal to 10 mm, in particular, have been identified as a significant risk factor for CRC development [5]. The removal of these polyps through procedures such as colonoscopies can prevent them from progressing into cancer. For early detection of polyps, the National Health Service (NHS) has set up the Bowel Cancer Screening Programme (BCSP) to offer screening for the detection of pre-cancerous growths, such as polyps [6].

The national BCSP is currently offered to those individuals aged 54-74 years, every two years. Those aged 75 years and above can opt back in on request. Individuals are given a faecal immunochemical test (FIT) kit, and those testing abnormal are then offered a colonoscopy, the gold standard test for CRC [6]. Patients diagnosed with polyps undergo polypectomy, and follow-up in such cases is referred to as post-polypectomy surveillance colonoscopy. Patients are typically placed on a surveillance schedule, with repeat colonoscopies at set intervals. However, those who have clear screenings may not undergo further testing for several years. Therefore, the efficacy of CRC surveillance is contingent upon accurate polyp detection during the initial colonoscopy, referred to as the “index” colonoscopy. Even with modern techniques and thorough procedures, some polyps are missed during the initial colonoscopy [7].

A study by Rutter and colleagues presented comprehensive surveillance guidelines endorsed by the British Society of Gastroenterology (BSG), the Association of Coloproctology of Great Britain and Ireland (ACPGBI), and Public Health England (PHE). These guidelines provided evidence-based recommendations on surveillance intervals and risk stratification, aiming to reduce the risk of CRC recurrence and optimise patient outcomes following polypectomy or CRC resection. These guidelines are currently implemented and followed by the national BCSP in the UK [4]. The rate of missed polyps during bowel cancer screening has been previously documented; however, a significant disparity exists regarding its prevalence rate [8, 9].

Despite the efficacy of BCSP, the occurrence of missed polyps raises concerns about the efficacy of the screening process. This study, therefore, aims to explore the frequency and causes of significant missed polyps and evaluate the implications for patient care. The findings of the present study will help decision-makers, enhance the effectiveness of the BCSP, and ultimately reduce the burden of CRC in the UK population.

## Materials and methods

Study population

A retrospective analysis of BCSP patients who, between January 2020 and March 2023, had undergone a primary colonoscopy, followed by either site checks or planned polypectomies, was conducted. Three hospitals were involved in the BCSP screening in Cheshire, UK: Countess of Chester Hospital, Leighton Hospital, and Macclesfield District General Hospital. Site checks were primarily done between three and six months following the index colonoscopy and again at 12 months after the index colonoscopy. Polypectomies were performed within 12 months of the index colonoscopy. A significant polyp was defined as a polyp >=10 mm.

The size of the polyp was measured following resection during histological analysis. The age, gender, and Boston Bowel Preparation score of patients were recorded. The Boston Bowel Preparation Scoring System scored the right, transverse, and left segments of the colon on a scale of 0 to three, for a total score out of nine. A score <=5 was poor, six to seven was good, and >=8 was excellent. Patients who had a normal result at index colonoscopy or a failed test were excluded. Furthermore, those with colorectal malignancies, inflammatory bowel disease, surgical referrals, and those who were undergoing symptomatic surveillance management were also excluded.

Colonoscopy procedure

Patients were given PLENVU® (Salix Pharmaceuticals, Bridgewater, NJ) bowel preparation before their procedure in either single dosing or split dosing. Procedures were conducted both in the morning and afternoon. Each procedure was performed by a BSCP-trained endoscopist. Any polyps detected during the index or subsequent procedures were recorded and removed at the time. In cases where this was not possible, these polyps were recorded and removed at a later procedure, and these polyps were not counted as missed polyps. The polyps detected were histologically classified as tubular adenoma (TA), tubular villous adenoma (TVA), or sessile serrated lesion (SSL), with or without low-grade dysplasia (LGD) or high-grade dysplasia (HGD). In addition to the size (in mm), the location of the polyps was also recorded (caecum, ascending colon, transverse colon, splenic flexure, descending colon, sigmoid colon, and rectum).

Polyp miss rate in the study sample

Polyps that were first detected during subsequent procedures, following the index colononoscopy, were classified as missed polyps. Subsequent procedures consisted of either full colonoscopies or flexible sigmoidoscopies. The missed polyp rate was calculated as (total number of missed polyps) / (total number of missed polyps + total number of polyps found on index colonoscopy). The significant missed polyp rate was calculated as (total number of significant missed polyps) / (total number of missed polyps + total number of polyps found on index colonoscopy).

Statistical analysis

For data analysis, IBM SPSS Statistics software, version 25 (IBM Corp., Armonk, NY), was used. Descriptive statistics were used to summarise patient demographics, polyp characteristics, and procedural data. Continuous variables, such as age, were reported as means and ranges, while categorical variables, such as polyp morphology and location, were presented as frequencies and percentages. Multivariable logistic regression analysis was employed to identify patterns and trends associated with missed polyps and significant missed polyps. P-values less than 0.05 were considered statistically significant.

## Results

Miss rate of colorectal polyps

**Figure 1:**
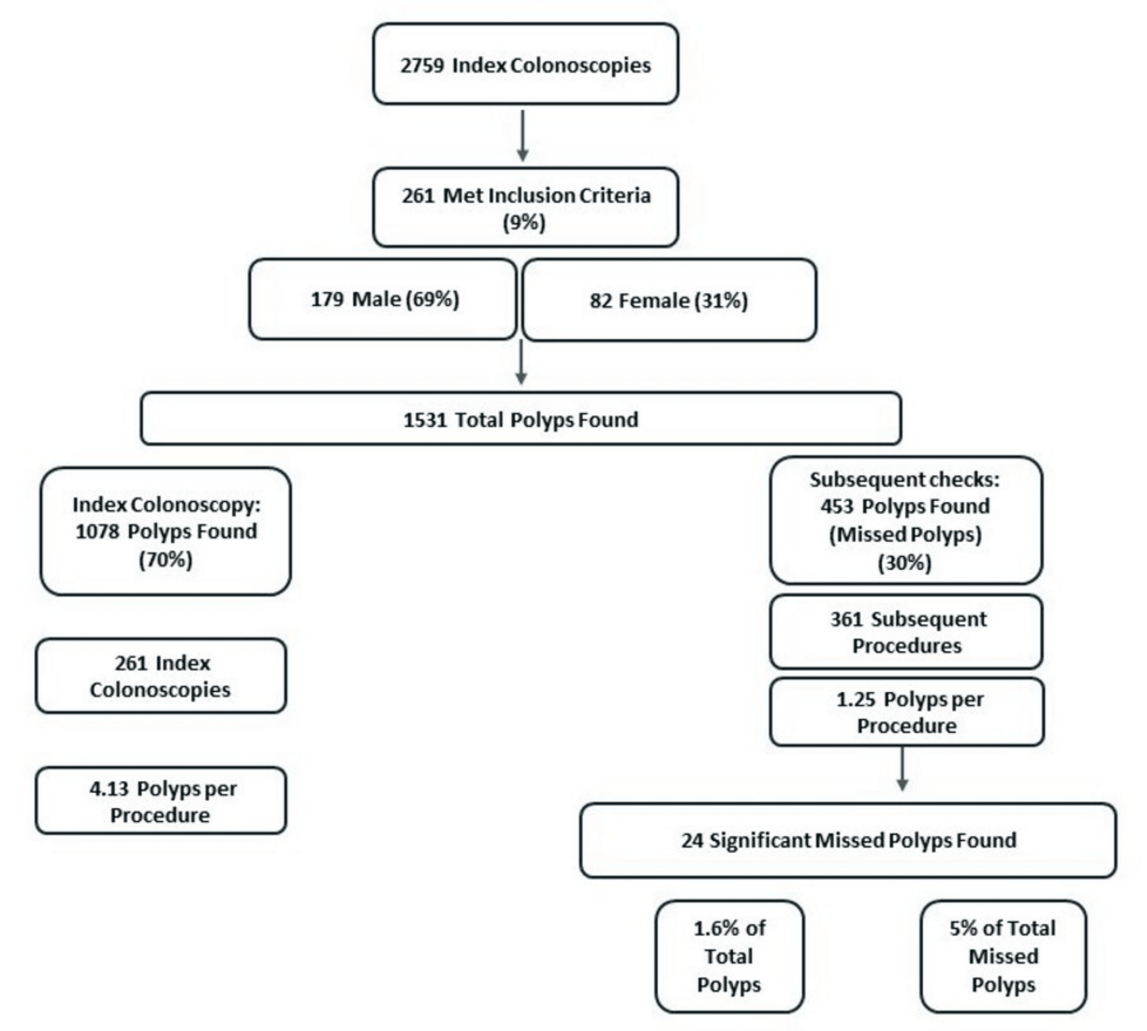
A flowchart displaying the breakdown of the results

Out of 2,759 patients (59% male), 261 (9.5%) met our inclusion criteria, and 23 (8.8%) of these had significant missed polyps. Of the 261 (179 male; mean age: 69 years; age range: 57-82 years), the missed polyp rate was 30% (453/1531 polyps). Of the 23 patients (17 male; mean age: 68 years; age range: 57-78 years), the significant missed polyp rate was 1.6% (24/1531). Of the missed polyps, 5% (24/453) were significant polyps (Figure 1). A total of 261 index procedures were carried out and 361 subsequent procedures; 75% (270/361 procedures) of the subsequent procedures were colonoscopies, and 25% (91/361 procedures) were flexis.

Location of significant missed polyps

A total of 71% of the significant missed polyps were found in the left of the colon (21% in the sigmoid colon, 21% in the rectum, 17% in the descending colon, and 16% in the splenic flexure) (Table 1).

**Table 1:** Analysis of significant missed polyps detected TA: tubular adenoma; TVA: tubular villous adenoma; LGD: low-grade dysplasia; SSL: sessile serrated lesion

Parameters	Patients with significant missed polyps, n= 23 (%)
Number of significant missed polyps	24
Location	
Ascending	2 (8%)
Transverse	2 (8%)
Descending	4 (17%)
Sigmoid	5 (21%)
Rectum	5 (21%)
Splenic flexure	3 (13%)
Caecum	3 (13%)
Morphology	
Flat	15 (63%)
Pedunculated	7 (29%)
Semi-pedunculated	2 (8%)
Size, mm	
10 mm	16 (67%)
11-19 mm	7 (29%)
20 mm	1 (4%)
Pathology	
TA with LGD	12 (50%)
TVA with LGD	10 (42%)
SSL	1 (4%)
Hyperplastic	1 (4%)

Characteristics of significant missed polyps

Out of the significant polyps found, 63% (15/24 polyps) were flat; 100% of the significant polyps found met the criteria to be classified as premalignant (50% TA LGD, 42% TVA LGD, 4% SSL, and 4% hyperplastic). The significant missed polyps ranged between 10-20 mm in size, with 66% (16/24 polyps) being 10 mm big (Table 1).

Characteristics of patients with missed polyps 

Men had a higher missed polyp rate of 22% (337/1531) compared to 7% (116/1531) in women (relative risk (RR) = 2.56, 95%CI: 2.1-3.13, p<0.0001). They also had a higher significant missed polyp rate of 1.1% (17/1531) compared to 0.4% in women (7/1531) (RR=2.41, 95%CI: 1-5.8, p<0.05) (Table 2).

**Table 2:** Gender differences in missed polyp rates

Characteristic	Men	Women	Relative risk (RR)	95% Confidence interval (CI)	p-value
Missed polyp rate	22% (337/1531)	7% (116/1531)	2.56	2.1-3.13	<0.0001
Significant missed polyp rate	1.1% (17/1531)	0.4% (7/1531)	2.41	1-5.8	<0.05

Factors associated with significant missed polyps 

In those patients identified as having significant missed polyps, during their index colonoscopy, 50% of them had bowel prep rated as ‘adequate/fair’. At the time of discovery of the significant missed polyp, 79% of them had bowel preparation rated as either ‘excellent’ or ‘good’ (odds ratio (OR) = 3.8, 95%CI: 1.07-13.5, p<0.05) (Table 3).

**Table 3:** Bowel preparation in patients with significant missed polyps

Bowel preparation rating	Index colonoscopies, n=24 (%)	At the time of significant polyp discovery, n=24 (%)	Odds ratio (OR)	95% Confidence interval (CI)	p-value
Excellent/Good	12 (50%)	19 (79%)	3.8	1.07 - 13.5	<0.05
Adequate/Fair	12 (50%)	5 (21%)	0.26	0.07-0.93	<0.05

Patients with >=2 polyps at the index were found to have a lower risk of missed polyps in subsequent procedures compared to those patients with just one polyp found at the index (RR = 0.69, 95%CI: 0.54-0.88, p<0.005). These patients were also at lower risk of a significant polyp being missed; however, the difference was not statistically significant (RR = 0.43, 95%CI: 0.13-1.39, p>0.05) (Table 4).

**Table 4:** Relationship between the number of index polyps and missed polyps

	No. of missed polyps, n=453 (%)	No. of polyps detected at the index colonoscopy, n=1531(%)
Patients with >=2 polyps at the index	411 (91%)	1019 (67%)
Patients with 1 polyp at the index	42 (9%)	59 (33%)

## Discussion

Missed polyps during colonoscopy remain a persistent challenge in CRC prevention. The findings of the present study showed that the rate of missed polyps is 30%. These findings are slightly higher compared to previous studies. For example, a review of six studies involving 465 patients reported a pooled missed polyps rate of 22% (95% CI: 19-26%) [8]. Similarly, another systematic review and meta-analysis by Zhao et al. reported a miss rate of 26% (95% CI: 23-30%) [10]. The significant missed polyp rate of 1.6% found in this study is comparable to the previous studies. For example, Shehadeh et al. reported a miss rate of advanced polyps of 1.7%. Their study included 232 patients [11]. Similarly, van Rijn et al. reported a miss rate of 2.1% for polyps > or =10 mm [8]. Missed polyps, particularly those larger than 10 mm, have the potential to progress to CRC, which underscores the importance of minimising the miss rates for these polyps [4,12,13].

Given that adenoma detection rate is a critical quality indicator in colonoscopy, the findings from this study suggest that even small improvements in detection rate could have a substantial impact on reducing the incidence of interval CRC. Corely et al., based on their analysis of 314,872 colonoscopies, reported that a 1% increase in adenoma detection rate could lead to a 3% reduction in fatal interval CRCs [12]. Moreover, the predominance of flat polyps among missed lesions (63%) is consistent with other studies that have identified flat morphology as a significant risk factor for missed polyps [14]. Kim et al., in their study, reported that flat or sessile polyps are more likely to be missed compared to pedunculated or sub-pedunculated polyps (adjusted OR, 3.62; 95% CI, 2.40-5.46) [7].

Flat polyps can be difficult to detect due to their subtle appearance and the challenges they pose in visualisation during colonoscopy. The findings that flat and sessile polyps were more likely to be missed are particularly concerning, given their association with higher malignancy potential, especially in the case of sessile serrated lesions (SSLs) [4]. Sessile serrated lesions are often subtle and can be mistaken for normal mucosa, yet they contribute significantly to the development of interval cancers. These findings suggest a critical need for enhanced detection techniques, including the use of artificial intelligence (AI)-based tools that have shown promise in improving the identification of subtle lesions during colonoscopy [15]. These findings highlight the importance of proper training for endoscopists, including in advanced imaging techniques that have been shown to improve the detection of flat and small lesions [16].

The finding that 71% of significant missed polyps were located in the left colon, particularly in the sigmoid and rectum, contradicts previous studies that have reported a higher miss rate in the proximal colon [15,17]. However, Leufkens et al., in their study, also reported that more than two polyps during colonoscopy and localisation in the left colon are the main factors that increased the risk of missed polyps [18]. The reasons for this could include suboptimal bowel preparation, shorter withdrawal times, and more complex bowel anatomy in these regions. It is also worth noting here that 25% of subsequent procedures were flexis, which could have influenced these results. The gender disparity observed in this study, with men having a higher missed polyp rate compared to women, is consistent with broader research that indicates men are at a higher risk of developing adenomas and advanced neoplasia [19].

McCashland et al. reported that men have a greater risk of polyps compared to women (OR = 1.5) [20]. This increased risk in men may be attributed to factors such as differences in bowel anatomy, hormonal influences, and lifestyle factors, including diet and smoking [21,22]. This finding suggests that CRC screening programs should consider gender-specific strategies. The study also highlights the significant impact of bowel preparation quality on polyp detection rates. Suboptimal bowel preparation has long been recognised as a major factor contributing to missed lesions, as it impairs mucosal visualisation and can obscure small or flat polyps [23]. The finding that 50% of significant missed polyps occurred in patients with 'adequate/fair' bowel preparation during their index colonoscopy suggests that even minimally acceptable preparation may not be sufficient to ensure high detection rates.

Optimising bowel preparation protocols have been shown to improve bowel cleanliness and increase the adenoma detection rate [24]. Additionally, patient education and adherence to preparation instructions are crucial, as studies have shown that patient compliance with preparation protocols is a key determinant of bowel cleanliness [25]. Interestingly, this study found that patients with two or more polyps identified during the index colonoscopy were less likely to have polyps missed in subsequent procedures. This contrasts with some previous studies, which have suggested that a higher number of polyps might be associated with an increased likelihood of missing additional lesions due to cognitive overload or time constraints [7,18].

Technological innovations and future directions

The findings of this study underscore the need for continued advancements in colonoscopy technology and technique. The integration of AI in colonoscopy is a promising area of development that could address some of the challenges highlighted in this study; AI-assisted colonoscopy systems have demonstrated significant potential in increasing polyp detection rates, particularly for small and flat lesions that are easily missed by human eyes [15]. These systems analyse the video feed in real-time, flagging potential polyps for the endoscopist to examine more closely, thereby reducing the likelihood of missed lesions. Furthermore, the implementation of quality assurance measures, such as regular monitoring of individual endoscopist performance and feedback on missed polyp rates, could enhance detection rates and reduce variability in performance. Adherence to established guidelines, such as those outlined by Rutter and colleagues, remains critical in maintaining high standards of care within CRC screening programs [4].

Limitations

There are certain limitations of the study as well, which should be kept in mind while interpreting the findings of this study. Firstly, as a retrospective review, the findings are limited to the specific subset of patients who underwent a site check or a subsequent procedure following the index colonoscopy. This means that the missed polyp rate calculated may not accurately represent the overall rate in the entire screening population, as only a small percentage of patients deemed at higher risk were included. Bringing back every patient initially screened to assess the miss rate in the entire group was not feasible, and the BCSP protocol dictates that only patients considered at the highest risk of colorectal cancer are followed up with additional procedures.

## Conclusions

In conclusion, the findings of the study revealed that approximately one-third of the polyps are missed during initial colonoscopies in BCSP. The study showed several key findings that can have clinical implications. For example, the majority of the missed polyps were located on the left side of the colon, with a notable portion being flat lesions, which are often more difficult to detect. Furthermore, men were more likely to have missed polyps compared to women. The quality of bowel preparation was identified as a critical factor, with many patients showing inadequate preparation during their first procedure but improved results in follow-up screenings. Patients who had multiple polyps detected during their initial colonoscopy were less likely to have missed polyps in subsequent procedures, a finding that has not been generally reported previously. The importance of the BCSP cannot be overstated; it is a cornerstone of CRC prevention in the UK. However, as this study demonstrates, there is a significant opportunity to maintain and enhance its effectiveness by addressing the factors that contribute to missed polyps.
